# Did place-based industrial policy promote regional economic growth? ---- Evidence from China

**DOI:** 10.1371/journal.pone.0283688

**Published:** 2023-04-04

**Authors:** Jianzhao Liu, Na Li, Xueli Du

**Affiliations:** School of Economics and Management, Tianjin Chengjian University, Tianjin, China; Shenzhen University, CHINA

## Abstract

Whether the place-based industrial policy promotes regional economic growth is a hot issue in the field of regional industrial economic practice. As a major national strategy in China, the Beijing-Tianjin-Hebei industrial coordinated development policy has been implemented more than 8 years. Verifying its effect on regional economic growth and revealing the policy action path will help to further optimize the policy implementation process through feedback. In this paper, the policy effect and its differentiation are empirically studied from ‘quality’ and ‘quantity’ respectively by establishing a growth model using the Dual Differences method. The results show that the Beijing-Tianjin-Hebei industrial coordinated development policy improves total factor productivity by 2.26% in terms of ‘quality’, and reduces GDP growth rate by 4.65% in terms of ‘quantity’. For the different region, the GDP growth rate increased by 1.28%, while total factor productivity decreased by 2.63% in Beijing, the GDP growth rate decreased by 3.17% and total factor productivity increased by 0.87% in Tianjin, and the GDP growth rate increased by 2.56% and total factor productivity increased by 1.58% in Hebei. The policy is mainly realized by fixed asset investment, capital deepening degree and enterprise scale expansion, while the effect of labor input, R&D investment and enterprise number is not significant. The policy is to give full play to the driving role of fixed asset investment such as "new infrastructure", increase investment in labor and research and development in the region, strengthen the construction of a competitive market environment, and stabilize the ‘quality’ and ‘quantity’ to further release policy dividends.

## 1 Introduction

In February 2014, the Beijing-Tianjin-Hebei Coordinated Development Major National Strategy of China was launched, a series of policies for coordinated industrial development have been successively promulgated and implemented (Table 1). The report of the 19th National Congress of the Communist Party of China clearly put forward ‘relocate non-essential functions of Beijing’s role to promote the Beijing-Tianjin-Hebei Coordinated Development, which means that adjusting the regional economic structure and spatial structure will make the strategic focus and implementation clearer. Up to now, the implementation of the policy has been more than seven years, the policy effect began to appear.

**Table 1:** List of representative policies Beijing-Tianjin-Hebei industrial coordination.

Year	Policy Name	Policy Issuing Agency	Key points of policy
2015	*Outline of the Plan for the Beijing-Tianjin-Hebei Region Coordinated Development*	Leading Group for Beijing-Tianjin-Hebei Coordinated Development of State Council of PRC	Introduce a package of industry-related plans and measures
2015	*Measures for sharing tax revenue from enterprises connected with industrial transfer in the Beijing-Tianjin-Hebei coordinated development*	Finance Bureau, Taxation Bureau	Specific measures for enterprises to share value-added tax, enterprise income tax and business tax in the places where they move in and out
2015	*The Special plan for scientific and technological innovation in the Coordinated Development of the Beijing-Tianjin-Hebei region*	Ministry of Science and Technology	Beijing Tianjin and Beijing Hebei jointly built 13 science and technology parks
2015	*A plan for the coordinated development of the Beijing-Tianjin-Hebei region*	National Development and Reform Commission, Ministry of Transport	Transportation support industries coordinated development
2016	*Opinions on providing judicial services and guarantee for the coordinated development of The Beijing-Tianjin-Hebei Region*	Supreme People’s Court	The industrial coordinated development is guaranteed by "coordinated judicature"
2017	*Relieve Non-capital function of Beijing*	Beijing Municipal Development and Reform Commission	Adjust the industrial structure and reduce the proportion of non-capital industries in Beijing
2017	*Promote coordinated development of The Beijing-Tianjin-Hebei region through industrial collaboration*	Hebei Development and Reform Commission	promote the formation of a rational division of labor among industries and a mechanism for linking upstream and downstream industries
2018	*Build a demonstration zone for Beijing-Tianjin-Hebei region Coordinated Development*	Tianjin Development and Reform Commission	Clear the functions of the pilot Free Trade Zone, Tianjin Port, and northern China’s core international shipping area

According to a report by the National Bureau of Statistics of China, the Beijing-Tianjin-Hebei region has seen positive changes in its industrial structure, regional industrial division pattern and industrial coordination within the region. By the end of 2020, the number of legal entities in the secondary and tertiary industries of Beijing, Tianjin and Hebei increased by 57 percent, 37 percent and 163 percent respectively compared with the end of 2013, there is 16,000 inter-provincial (municipal) industrial activity units in the Beijing-Tianjin-Hebei region, up 180 percent from the end of 2013. From the perspective of innovation input, the R&D investment intensity of Beijing-Tianjin-Hebei region reached 3.36% in 2020, an increase of 0.37% compared with 2013. The R&D investment intensity ratio of Beijing-Tianjin-Hebei Region also changed from 5.86:2.93:1 in 2013 to 4.45:1.89:1 in 2020, narrowing the gap between the three regions. However, some studies have found that during the same period, the per capita GDP of the Beijing-Tianjin-Hebei region was 17% higher than the national average, significantly lower than the 28% in 2014. The standard deviation of per capita GDP has also increased from 2.96 in 2014 to 3.99, reflecting the lower-than-expected effect of industrial coordinated development in the region.

The inconsistency between the above statistics and the research reflects the complexity of the research on policy effects. It is difficult to judge whether industrial policy can promote industrial growth and regional development, but it is crucial. Up to now, there is no consensus on whether industrial policy is effective. Some studies have found no positive effects of industrial policy [1–4]. Some studies have even found adverse effects of industrial policy [5–7]. There are also many studies showing that industrial policy can promote industrial development [8–14]. There are also some studies believe that the effectiveness of industrial policy needs certain conditions [15–20].

Existing studies on the effects of industrial policies mostly focus on infrastructure, industrial investment, spatial distribution, transportation, enterprise innovation and other aspects, but there is no consensus on the effectiveness of industrial policies. On the one hand, the effect of industrial policy is usually realized in an indirect way, and often does not take a single project or enterprise as the policy object [21–23], but is implemented in coordination with various means of policy intervention [24–27]. On the other hand, industrial policy also needs to be based on the specific socio-economic environment and institutional system on which it depends [28–31]. Therefore, policy evaluation will face various difficulties such as multiple policy overlay and external environmental constraints. However, Buendia (2005) and Pinder (2017) still revealed the causal relationship and internal relationship between industrial policy and its effect [32, 33]. Boekholt (2003) also found that the effect of industrial policy not only acts on a certain type of cluster industry, but also acts on other industries through spillover effect [34]. Anusha Seetharaman et al. (2003) analyzed the economic benefits generated by freight transport policy in the transportation plan and believed that the policy could effectively promote the output growth of the freight industry [35]. Fay et al. (2013) assessed green industry policy as necessary to support the development of key new technologies and sectors, but with caution at the dual risks of market and governance failure [36]. Kemp et al. (2017) also outlined the possibilities for green industrial policies in developing countries [37]. Gruber et al. (2019) pointed out that digital industry policies should take into account the new opportunities brought about by digitalization and strengthen the intangible asset base along the digital economy value chain [38].

The most commonly used measurement standard to evaluate the effect of industrial policy is total factor productivity, but in the actual research process, due to different research objects and research paths, the conclusions obtained by scholars are also inconsistent. Hsieh & Klenow (2009) found that the stagnation of manufacturing total factor productivity was largely attributed to resource mismatch within the industry [39]. Criscuolo et al. (2012) used business data to study the positive impact of the UK’s investment subsidy scheme on employment, investment and net entry. However, contrary to our study, they had no effect on TFP [40]. Aghion et al. (2015) used government subsidies, tariffs and other tools to measure industrial policies and found that industrial policies could improve total factor productivity in competitive markets [41]. Zhang Guofeng (2019) found that the growth effect of the development zone policy and the agglomeration economy was very obvious, which significantly improved the productivity growth of enterprises [42]. Syverson (2011) summarized the source of industrial productivity as internal effect and inter-firm resource relocation effect [43]. Lucchese et al. (2016) analyzed the tools used in Italy’s industrial and innovation policy and argued that the likelihood of rebuilding Italy’s productive capacity depended largely on the formulation of new industrial policies [44]. Gebrewolde, Tewodros, and James Rockey (2022) used data from Ethiopian Manufacturing Enterprises, it was demonstrated that economic growth priority policies in specific regions was unsuccessful, and overall there was no substantial increase in productivity [45]. Alder et al. (2016) Believed that the establishment of China’s special economic zones had had a huge positive impact on the economy of the city where it operates [46].

Whether industrial policy effectively promotes regional economic growth is a key issue in the field of regional industrial economic practice, but there are few literatures that adopt rigorous quantitative methods. It is a complex research proposition that the existing research results show that the effects of industrial policy are positive and negative. Busso, Gregory & Kline (2013) believed that the decision of whether to adopt policy intervention, all the policy tools considered and the intervention effect depended on the local economic environment [47]. This conclusion is reasonable to some extent, but it also means that research on specific policy effects can only be carried out under specific conditions. In view of this, this paper mainly explores the effects of the Beijing-Tianjin-Hebei Industrial Coordinated Development Policy from the following aspects: First, whether the policy promotes regional economic growth; Second, has the policy narrowed the gap between the regions and its surrounding areas? Third, what is the mechanism that the policy effect is generated? In view of these three problems, this paper uses the dual difference method to measure the effect of the Beijing-Tianjin-Hebei Industrial Coordinated Development Policy on the economic growth and the Total Factor Productivity of the industry, and further analyze the incentive effect restricting the economic development of the region.

As a key area of the Beijing-Tianjin-Hebei Coordinated Development Major National Strategy of China, more solid econometric evidence is needed to determine whether the policy implementation has promoted regional development. Scientifically evaluating the effect of industrial policies, and then feedback regulation and optimization of policies, will promote the endogenous self-reinforcement of policies and long-term sustainable promotion of regional industrial development. In this paper, the dual difference method is used to explore the regional economic growth effect of the Beijing-Tianjin-Hebei Industrial Coordinated Development Policy, and reveal the action path of the policy, so as to provide optimization and improvement basis for the implementation of the Beijing-Tianjin-Hebei Coordinated Industrial Development Policy.

## 2 Empirical strategy

### 2.1 Model

The dual difference method is often used to study policy issues since Ashenfelter, Orley C., and David Card (1984) first applied this method [48]. The core idea of this method is to set up two groups, the Treatment Group and the Control Group. The Treatment Group is affected by the policy at *t* time point, and the Control Group is not affected by the policy at *t* time point, that is, the two groups are different in policy treatment. The effect of policy implementation is evaluated by comparing the output changes of treatment group and control group before and after policy implementation.

Mayer (1994) made an in-depth study of the dual difference method. He set a treatment group that began to be affected by the policy at *t* time point, and assumed that the group had observable changes in economic behavior before and after the implementation of the policy. Thus, the policy effect of the Treatment Group could be obtained through the following model:

$$y_{it}=\alpha +\beta d_{t}+e_{it}$$ (1)

Where, *y*_*it*_ is the output of representative individual *i*(*i* = 1,⋯,*N*) in the processing group at time *t*(*t* = 0 *or* 1), *d*_*t*_ is a dummy variable; the value is 0 when *t* = 0; the value is 1 when *t* = 1; *e*_*it*_ is the error term whose variance changes with time. When *E*[*e*_*it*_|*d*_*t*_] = 0, *β* can be used to measure the effect of policy changes. The value of *β* can be estimated by Eq (1), or can be obtained by calculating the difference between the average output before and after the time *t*.

Differential causes potential impact factor identification problems when evaluating policy effects. Because the regional output change at the time *t* is not only determined by the policy, but also may be affected by other factors. Based on this consideration, the dual difference method introduces other influencing factors that may cause output changes into the effect estimation model as control variables. Compared with the Treatment Group, the output changes of the Control Group are only affected by control variables, thus effectively separating the disturbance of potential influencing factors. Therefore, the dual difference model evolves into the following form:

$$y_{it}^{j}=\alpha +\beta_{1}d_{t}+\beta_{2}d^{j}+\beta d_{t}^{j}+e_{it}^{j}$$ (2)

Where *j* represents two groups, *j* = 1 is Treatment Group and *j* = 0 is Control Group. *d*^*j*^ is a dummy variable, its value is 1 when *j* = 1, and when *j* = 0, its value is 0. $d_{t}^{j}$ is also a dummy variable and the value is 1 When *t* = *j* = 1, otherwise its value is 0. *β* is the key observation coefficient, which can directly reflects the policy effect. *β*_1_ is the variation of output over time between the Treatment Group and the Control Group without the influence of policy, while *β*_2_ represents the difference between the two groups without the influence of time change. An important prerequisite for the use of the Dual Difference Model is that when *β* = 0, there is no policy change impact, the economic behavior of the Control Group and the Treatment Group has the same time trend.

In addition, in order to control individual differences and improve the effectiveness of estimation, this paper added control variables and finally set the regression model as:

$$y_{it}^{j}=\alpha +\beta_{1}d_{t}+\beta_{2}d^{j}+\beta d_{t}^{j}+Z_{it}\delta +e_{it}^{j}$$ (3)

Where, *Z*_*it*_ is the set of control variables affecting other characteristics of economic individuals, and *δ* represents the influence degree of each control variable on the explained variable.

Since the implementation of the Beijing-Tianjin-Hebei Industrial Coordinated Development Strategy, the region has experienced significant economic growth. However, how much of this economic growth is caused by the policy? In view of this, the application of dual difference method can effectively separate the impact of economic cycle disturbance on the regional economic growth, making the evaluation of the policy effect more objective and scientific.

In evaluating the "quantity" changes brought by the Beijing-Tianjin-Hebei Industrial Coordinated Policy, this paper adopts the output growth model as follows:

$$\Delta {gdp}_{it}=\log\left({gdp}_{it}/{gdp}_{it-1}\right)$$

$$=\alpha_{0}+{\alpha \Delta gdp}_{it-1}+\beta_{1}d_{2014}+\beta_{2}d^{j}+{\beta d}_{2014}*d^{j}X\beta +Z_{it}\delta +e_{it}^{j}$$ (4)

Where, *Δgdp*_*it*_ is explained variable, reflecting regional GDP growth rate. The growth model is a dynamic panel model because the lag period of the explained variable is introduced as the explanatory variable. The time dummy variable is *d*_2014_ (*d*_2014_ = 0 before 2014, and *d*_2014_ = 1 after 2014), and *d*^*j*^ is the group dummy variable (Within the Beijing-Tianjin-Hebei synergy region *d*^*j*^ = 0, outside industry in the Beijing-Tianjin-Hebei synergy region *d*^*j*^ = 1). The control variables are introduced to solve the heterogeneity between the Treatment Group and the Control Group which used to explain the growth rate of industrial output in the Beijing-Tianjin-Hebei region, including the growth of capital input, R&D input, labor input and market competition.

At the same time, in order to investigate whether the policy has brought about "quality" change, this paper builds a regression model of industrial productivity change:

$$TFP_{it}=\alpha_{0}+\alpha_{1}TFP_{it-1}+\beta_{1}d_{2014}+\beta_{2}d^{j}+\beta d_{2014}*d^{j}+Z_{it}\delta +e_{it}^{j}$$ (5)

Where, *TFP*_*it*_ is the total factor productivity, the control variables *Z*_*it*_ is used to explain the industrial productivity in the Beijing-Tianjin-Hebei region, including the growth of R&D investment, capital deepening, market competition and enterprise size. The meanings of other parameters are basically consistent with the above formula.

### 2.2 Treatment Group selection

In this paper, the Beijing-Tianjin-Hebei Industrial Coordinated Policy is taken as a "natural experiment". Beijing city, Tianjin city and 11 prefecture-level cities in Hebei province are selected as the Treatment Group, and the prefecture-level cities in Shanxi, Henan and Shandong province around Beijing-Tianjin-Hebei are selected as the Control Group. Because the Control Group and the Treatment Group both belong to the Bohai Economic Circle with close spatial proximity and economic connection, besides the economic growth trend of the two groups before the implementation of the Beijing-Tianjin-Hebei coordinated industrial development policy was basically the same, the dual difference method can effectively identify the effect of the industrial coordination policy.

The introduction of a series of control variables can effectively solve the heterogeneity between the Treatment Group and the Control Group. This paper selects the control group following two principles: First, there is no national economic zone planning. If the Control Group is only selected based on the similar level of economic development, such as the "Yangtze River Delta" and "Pearl River Delta" national economic zones as the control group, the bias of the policy impact of these economic zones cannot be removed, and the net effect of the policy cannot be effectively assessed. Looking at the policy implementation areas of China’s economic zone, after excluding the "western development", "Northeast revitalization", "Yangtze River Delta", "Pearl River Delta", "Guangdong-Hong Kong-Macao Greater Bay Area" and other policy areas, Shanxi, Shandong and Henan are better reference object to compare with Beijing, Tianjin and Hebei. Second, before the implementation of the Beijing-Tianjin-Hebei industrial coordinated policy, the GDP growth rate and total factor productivity of the Control Group were basically consistent with Beijing-Tianjin-Hebei. A necessary condition for the use of DID is that the Treatment Group and the Control Group have the same trend before the policy change [49]. Before the implementation of the Beijing-Tianjin-Hebei industrial coordination policy, the explained variables of the study Treatment Group and Control Group should have the same changing trend, so as to directly reflect the net effect of the policy on the economic growth of The Beijing-Tianjin-Hebei region. Therefore, in order to verify the applicability of DID model and the rationality of the selection of control group, the same trend test is conducted on the GDP growth rate and total factor productivity of the treatment group and the control group respectively.

**Fig 1:**
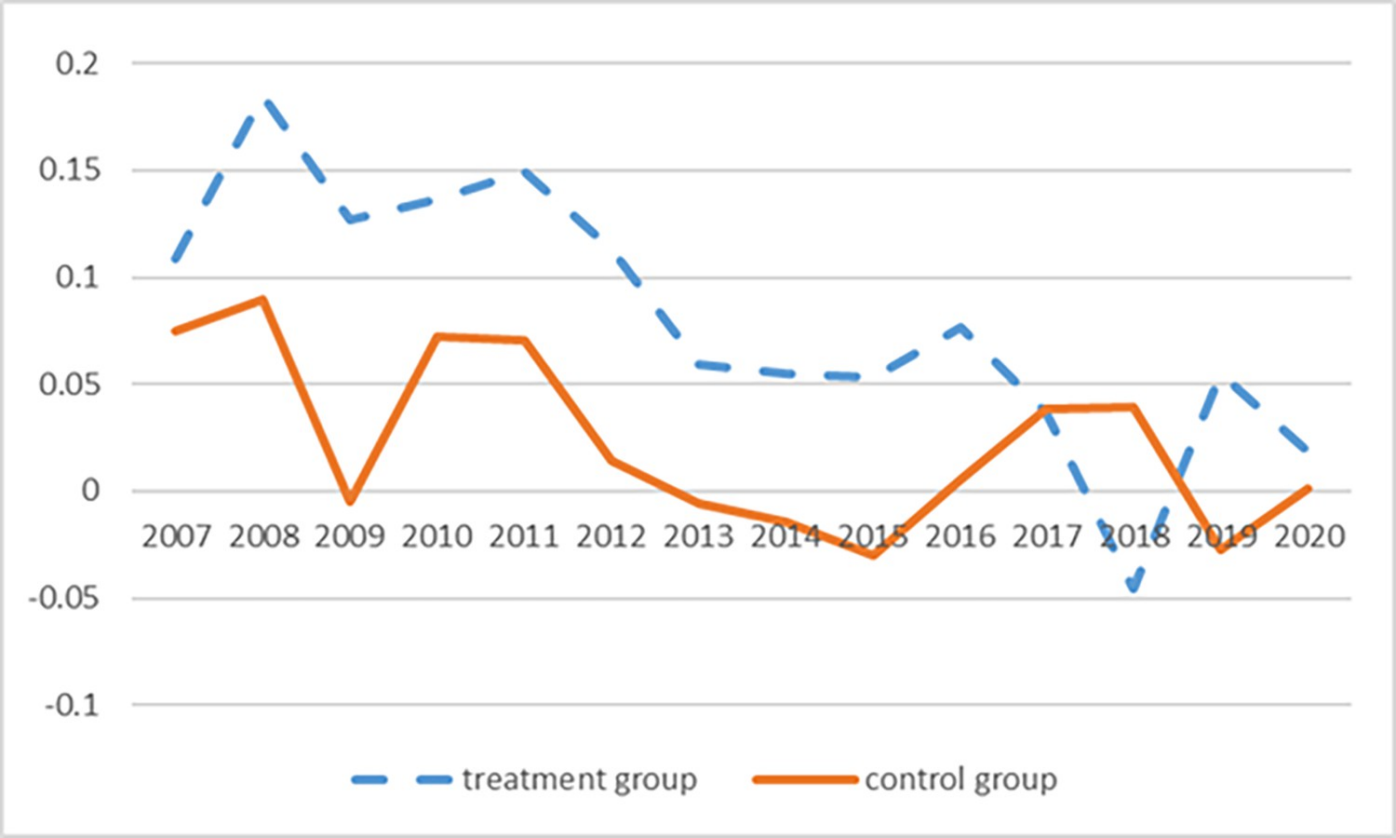
The average GDP growth rate of the Treatment Group and the Control Group.

Before 2014, the Treatment Group and the Control Group showed a similar trend, and they basically maintained the same trend from 2007 to 2014. However, after 2014, the GDP growth rate of the Treatment Group and the Control Group became a significant difference, and the two groups gradually converged, indicating that the regional economy tended to converge under the effect of industrial policies (Fig 1). Up to now, the total factor productivity change range is not significant. Through its trends the status of the Treatment Group much better than Control Group, but in 2014, the policy has more obvious improvement. Therefore, the rate of GDP growth and total factor productivity in the Control Group and Treatment Group before the policy implementation is basically consistent, so using the DID model to examine the policy effect, which confirms to the premise of the same trend hypothesis.

In order to solve the rationality of Control Group, this paper conducted paired sample T test on GDP growth rate and total factor productivity of Shanxi, Shandong, Henan and Beijing, Tianjin and Hebei from 2007 to 2013 (before the implementation of industrial coordination policy), and compared to see whether there were significant differences between them (Table 2).

**Table 2:** Paired sample T test.

Paired sample test 95% confidence interval for difference							
	mean	The standard error of the mean	lower limit	upper limit	t	df	Sig(Double tail)
**Beijing-Tianjin-Hebei—Jinluyu** **GDP growth rate**	0.308	1.1506103	-1.7008	1.08500	-.541	6	0.608
**Beijing-Tianjin-Hebei—Jinluyu** **Total factor productivity**	0.165	0.022577	0.1437	0.1854	19.281	6	0.433

It can be seen that Sig(double-tail) values of pairing test for Shanxi, Shandong, Henan and Beijing-Tianjin-Hebei are all greater than 0.05, which means there is no significant difference between GDP growth rate and total factor productivity. Therefore, the selection of control group is ok.

### 2.3 Variable and data

In this paper, the policy effect of Beijing-Tianjin-Hebei Industrial Coordinated Development is measured from the aspects of output growth and total factor productivity. Before model estimation, total factor productivity should be calculated. Considering the endogenous of production function, LP method in semi-parametric method will be used to calculate total factor productivity, and the total factor productivity is obtained by the following Cobb-Douglas production function:

$$y_{it}=\beta_{0i}+\beta_{li}l_{it}+\beta_{ki}k_{it}+\beta_{mi}m_{it}+\omega_{it}+\eta_{it}$$ (6)

Where *y*_*it*_, *l*_*it*_, *k*_*it*_ and *m*_*it*_ are respectively *gdp* added value, labor input, capital input and net inflow of goods and services, which are adjusted by consumer price index, industrial producer purchase price index and fixed asset investment price index. The LP method assumes that under the given *k*_*it*_ circumstances, *m*_*it*_ is a monotone increasing function of *ω*_*it*_, and is expressed as *m*_*it*_ = *m*_*t*_(*k*_*it*_, *m*_*it*_). Its inverse function can be expressed as *ω*_*it*_ = *h*_*t*_(*k*_*it*_, *m*_*it*_), the function that productivity can be represented by capital input and net inflow of goods and services, which can be obtained by substituting this function into Formula (6).


$$y_{it}=\beta l_{it}+\phi_{t}(k_{it},m_{it})+\varepsilon_{it}$$ (7)

$\phi (k_{it},m_{it})=\beta_{k}k_{it}+\beta_{m}m_{it}+h_{t}(k_{it},m_{it}),$ We estimate the *β*_*l*_ and *ϕ*_*t*_, move *l*_*it*_ to the left side of the model, and assume that the change of productivity conforms to a first-order Markov process, so get:

$$\omega_{it}=E(\omega_{it}|\omega_{it-1})+\xi_{it}=g(\omega_{it-1})+\xi_{it}$$ (8)

There are:

$$y_{it}-\beta_{l}l_{it}=\beta_{k}k_{it}+\beta_{m}m_{it}+\omega_{it}+\varepsilon_{it}=\beta_{k}k_{it}+\beta_{m}m_{it}+g(\omega_{it-1})+\xi_{it}+\varepsilon_{it}$$

$$=\beta_{k}k_{it}+\beta_{m}m_{it}+g(\phi_{t-1}-\beta_{k}k_{it-1}-\beta_{m}m_{it-1})+\xi_{it}+\varepsilon_{it}$$ (9)

In the above formula, *g*(*ω*_*it*−1_) is the relevant function expression of *ω*_*it*−1_, *ξ*_*it*_ is the information set between period *t*−1 and *t*, which is not related to *k*_*it*_ and *ε*_*it*_, so it can be regarded as one of the Torque conditions to identify the validity of the model. There is a decision variable *m*_*it*_ of industry in the period *t*, which are not correlated with *ε*_*it*_, but correlated *ξ*_*it*_, therefore, the net inflow of goods and services lagging one period *m*_*it*−1_ is selected as the instrumental variable. $g(\phi_{t-1}-\beta_{k}k_{it-1}-\beta_{m}m_{it-1})$ is represented by polynomial $\phi_{t-1}-\beta_{k}k_{it-1}-\beta_{m}m_{it-1}$, and GMM estimation is used to obtain effective estimates *β*_*k*_ and *β*_*m*_. At this point, the two-stage estimation is completed, and values of *β*_*l*_、*β*_*k*_ and *β*_*m*_ are estimated. Thus, the effective estimate of industrial total factor productivity *ω*_*it*_ can be further obtained.

In this paper, the data are from statistical yearbooks and statistical bulletins of various provinces and cities. Panel data from 2007 to 2020 are selected as samples, 2006 is taken as the base period for regional GDP, fixed asset investment, net inflow of goods and services are exponentially deflated to obtain the GDP growth rate and total factor productivity of the Treatment Groups and Control Groups from 2007 to 2020. In addition to the Beijing-Tianjin-Hebei Industrial Coordinated Development Policy affecting regional economic development, there are still some other exogenous factors, so it is necessary to control the interference of these factors, and the introduction of control variables can effectively deal with the heterogeneity between the Control Group and the Treatment. Drawing on the research of Sun Xiaohua and Guo Xu (2015), Zhang Guojian et al. (2019) [50, 51], in this paper, the following control variables are selected from the model representing the growth rate of industrial added value with "quantity" change: the growth rate of fixed asset investment is used to represent the growth rate of capital input (*Δk*). Measure the growth of labor input by the total wage of employed persons at the end of the year (*Δl*). The growth rate of R&D expenditure at the end of the year is expressed as the growth rate of R&D investment (*Δrd*). Market competition is measured by the number of enterprises in the number of basic units (*num*).The following control variables are selected from the model representing the total factor productivity of "quality" change: the growth rate of R&D expenditure at the end of the year represents the growth rate of R&D investment (*Δrd*). Capital labor ratio is used to represent the degree of capital deepening (*k*/*l*). Measure the degree of industrial scale by the total output value of industrial enterprises above designated size (scale). Measure market competition by the number of enterprises in the number of basic units (*num*).

## 3 Empirical research

### 3.1 Descriptive statistics

By measuring the GDP growth rate and total factor productivity of the Treatment Group and the Control Group from 2007 to 2020 (Table 3), it can be found that the average annual GDP growth rate of the two groups has shown a significant downward trend in the 13 years since 2007, but the total factor productivity has changed little, which may be caused by China’s economy Starting from high-speed development to high-quality development after 2013, it is difficult to see a big increase in total factor productivity in the short term. Since the implementation of the Beijing-Tianjin-Hebei Coordinated development strategy, the list of banned new industries has been implemented, general manufacturing enterprises have withdrawn, and some regional specialized markets in Beijing have been relieved. In order to control serious atmospheric pollution, Beijing-Tianjin-Hebei has eliminated a lot of traditional energy intensive high pollution discharge capacity, meanwhile, the pace of collaborative innovation in Beijing, Tianjin and Hebei has accelerated, and some science and technology innovation parks have initially taken shape. These policies and measures have accelerated the transformation process of the regional economic development model to quality improvement model, but still needs time to get significant results. Judging from the differences between different groups, there is an imbalance in economic development and industrial development between the treatment group and the control group. However, under the guidance of regional policies, regional mutual coordination, and industrial cooperation accelerated, and the gap began to narrow.

**Table 3:** Average values of explained variables in each year.

Year	GDP growth rate		Total factor productivity	
	Treatment group	Control group	Treatment group	Control group
2007	0.1706179	0.0810053	1.252261	1.110907
2008	0.1875414	0.0890832	1.281519	1.123986
2009	0.1284302	-0.0043817	1.241116	1.040785
2010	0.1382718	0.0723453	1.274801	1.131516
2011	0.1525044	0.0701292	1.291594	1.125926
2012	0.1149814	0.0139032	1.264272	1.074701
2013	0.060702	-0.0058884	1.222921	1.068924
2014	0.0564973	-0.0149177	1.242807	1.072604
2015	0.0551235	-0.0295301	1.266623	1.073822
2016	0.0786989	0.0057397	1.31825	1.128149
2017	0.0367011	0.0377932	1.250141	1.146809
2018	0.0405621	0.0384309	1.272922	1.163830
2019	0.05459332	-0.02727699	1.281394	1.178394
2020	0.01842486	0.001211914	1.290018	1.179736

To intuitively compare the differences between the two groups, Table 4 lists the descriptive statistical results of variables in the treatment group and the control group respectively. It can be seen that the average GDP growth rate of the treatment group was as high as 8.06% which maintain a high growth rate, and the gap between regions was significantly reduced from 2007 to 2020. The average output growth rate of the control group was slightly lower, 2.31%, and the difference between regions was slightly larger than that of the treatment group. Similarly, the total factor productivity of treatment group was higher significantly (1.036) than control group (1.027). In terms of control variables, the average growth rate of fixed asset investment and total labor wage in all research samples reached about 15%, and the growth rate of R&D investment reached more than 20%, the growth rate of the treatment group was still higher than that of the control group over all. The number of enterprises in the treatment group was nearly twice higher than control group, and the capital labor was also significantly higher than that in the control group, reflecting the increasingly high capital intensity attribute of the region. In addition, the total industrial output value between regions varies greatly, with the treatment group at 50.769 billion yuan, which is nearly three times of the control group, and indicates that the economic gap within the capital is still large. The reason is that the industrialization of the Beijing, Tianjin and Hebei is at different stages, the difference between capital, land, labor factor and output is significant, and the level of market economic development is uneven, and the number of large state-owned enterprises is large, and the development of the smaller private economy is insufficient.

**Table 4:** Descriptive statistics of variables.

Variable	Unit	Treatment Group		Control Group	
		Mean	Standard Deviation	Mean	Standard deviation
*Δgdp*	%	10.72791	0.0627229	2.86619	0.0686704
*tfp*	——	1.26421	0.1505415	1.09983	0.0821791
*Δk*	%	15.40753	0.1456756	16.63133	0.2374516
*Δl*	%	14.19221	0.0962733	7.77005	0.164481
*Δrd*	%	22.94273	0.2677474	21.6633	0.347394
*num*	Ten Thousand	7.365637	8.884222	3.960044	3.818781
*k*/*l*	Ten Thousand Yuan per person	35.08396	22.66949	24.99772	12.0059
*scale*	One Hundred Million Yuan	50.769	51.32315	17.33779	15.11639

### 3.2 Regression result

In this paper, OLS and GMM estimation methods are respectively adopted. Using GDP growth rate and total factor productivity as explained variables, and regression results as shown in Model (3) and Model (5) (Table 5) are obtained. In terms of the validity of the estimated results, the P value of AR (2) in model (4) is 0.064, and the P value of AR(3) in model (5) is 0.0170, indicating that the null hypothesis is accepted at the significance level of 10%, and the error term of model (4) and (5) meets the condition of no sequence correlation. In addition, Sargan values of both model (4) and model (5) after regression reach 1.00, indicating that the selected tool variables are effective. By comparing the fitting results of OLS and GMM, it can be found that the fitting coefficients of most control variables are significant at 1% level by using GMM regression, while the significance of each variable in OLS regression results is generally weak, especially the total factor productivity results are unsatisfactory. It can also be found that the coefficients of instrumental variables are significant at the level of 1%, and the coefficient of GDP growth rate lagging behind the first period is significantly positive (0.30) at the level of 1%, indicating that the national production is maintaining a sustained high-speed growth, and the GDP lagging behind the first period has a positive effect. The coefficient of TFP lagging one period is significantly positive (0.28) at 1% level, indicating that productivity improvement has a certain time lag, and the first-order lagging productivity has a positive effect on productivity improvement in the current period. By comparing the fitting results of the two estimation methods, GMM is more scientific and effective than OLS, and it can also solve the endogeneity problem in the model.

**Table 5:** Model regression results.

Explained variable: GDP growth rate			Explained variable: total factor productivity		
Model	(3)	(4)	Model	(3)	(5)
Method	OLS	GMM	Method	OLS	GMM
*L*.*Δgdp*		0.30253*** (4.19)	*L*.*tfp*		0.28012*** (2.17)
*d*_2014_	-0.04951*** (-8.13)	-0.02347*** (-2.70)	*d*_2014_	-0.01004 (-1.21)	0.00024 (0.01)
*d* ^*j*^	0.08179*** (10.86)	0.09871*** (2.98)	*d* ^*j*^	0.09770*** (10.48)	0.03696*** (0.74)
*d*_2014_*d^*j*^	-0.02203* (-1.86)	-0.04646*** (-2.09)	*d*_2014_*d^*j*^	-0.02409 (-1.62)	0.02262*** (0.32)
*Δk*	-0.07144*** (-7.29)	-0.10109*** (-7.31)	*Δrd*	0.00424 (0.47)	0.00084 (0.07)
*Δl*	0.03949** (2.37)	-0.00841 (-0.53)	*k*/*l*	-0.00003 (-0.12)	-0.00060*** (-0.65)
*Δrd*	0.00384 (0.52)	-0.00123 (-0.14)	*scale*	0.00172*** (10.85)	0.00168*** (2.25)
*num*	-0.0010** (-2.17)	-0.00126*** (-0.52)	*num*	0.00535*** (6.60)	0.00390*** (1.16)
*constant*	0.05866*** (11.96)	0.04137*** (3.42)	*constant*	1.0523*** (159.58)	0.76309*** (5.55)
*observations*	812	754	*observations*	812	754
*Abond test f AR*(1)	0.0000		*Abond test f AR*(1)	0.0033	
*Abond test f AR*(2)	0.0647		*Abond test f AR*(2)	0.5263	
*Abond test f AR*(3)	0.2695		*Abond test f AR*(3)	0.0170	
*sargan*	1.0000		*sargan*	1.0000	

Note: OLS estimation results are t-values in brackets

GMM estimation results are z-values in brackets

***, ** and * are significant at the level of 1%, 5% and 10% respectively.

The regression results of model (5) show that: compared with Shanxi, Henan and Shandong around Beijing-Tianjin-Hebei, in terms of ‘quality’, the coefficient of productivity effect (*d*_2014_*d^*j*^) of Beijing-Tianjin-Hebei Industry Coordinated Development Policy is significantly positive at the level of 1%. That is, the policy has a significant effect on promoting the total factor productivity, and the total factor productivity has increased by 2.26%. However, in terms of quantity, the effect of the Beijing-Tianjin-Hebei coordinated industrial development policy on GDP growth is significantly negative, only -4.65%. The following is a further study of the specific realization path of the effect of the Beijing-Tianjin-Hebei Coordinated Industrial Development Policy through the dual difference estimation of each control variable. The regression results of model (5) show that the regression coefficients of the enterprise production scale (*scale*) and number of enterprise (*num*)were significantly positive, indicating that its role in improving productivity is significant, and it also means that market competition has an important impact on total factor productivity, and the industrial concurrence and cooperation brought by a certain degree of competition is benign to the industrial development. According to “Measures for sharing tax revenue from enterprises connected with industrial transfer in the Beijing-Tianjin-Hebei coordinated development” published by the Ministry of Finance, the value-added tax and business tax of enterprises in the Beijing-Tianjin-Hebei region will be divided 50/50 in the places where they move to and from has effectively promoted the relocation of non-capital functions of Beijing and the directional transfer and agglomeration of industries, and the scale structure and spatial distribution of industries have been improved. The regression coefficient of capital-labor ratio (*k*/*l*) is significantly negative, indicating that the traditional practice of improving productivity by means of capital deepening has been reversed. A large amount of capital input has not changed the actual effect of its low efficiency, and the marginal income of capital input is decreasing year by year, which produces resistance to the improvement of productivity. As a result, the Beijing-Tianjin-Hebei industrial Coordinated Policy optimizes the direction of capital investment through voluntary transfer of subsidies and other policies, shifting from flood investment to targeted investment. The growth rate of R&D expenditure (*Δrd*) is positive but not significant, which may be application and industrialization of achievements are not high, and the effects of production mode, organization mode and technological innovation are difficult to be reflected in a short time, although the intensity of innovation investment is adequate. In order to further explore the realization path of Beijing-Tianjin-Hebei Industrial Coordination Policy effect, the dual difference method is continued to be used to investigate the effect of industrial coordination policy implementation on each control variable in the determined model. Table 6 lists the estimation results of the difference of each control variable.

**Table 6:** Difference in differences estimation results of control variables.

variable	Control groups 2007–2013	Process groups 2007–2013	Difference	Control groups 2014–2020	Process groups 2014–2020	Difference	DID
*Δk*	0.2209261 (0.23584)	0.1933293 (0.15983)	-0.027597 (0.0760)	0.0707408 (0.20880)	0.0853809 (0.08029)	0.0146401 (0.1285)	0.042237 (0.0525)
*Δl*	0.1120625 (0.19026)	0.1890717 (0.07460)	0.0770092 (0.1157)	0.0175668 (0.07380)	0.0594104 (0.07126)	0.0418436 (0.0025)	-0.035166 (0.11312)
*Δrd*	0.2756386 (0.40724)	0.2894711 (0.30198)	0.0138325 (0.1053)	0.1133732 (0.15897)	0.1243508 (0.14489)	0.0109776 (0.0141)	-0.0028549 (0.09118)
*num*	2.901939 (2.29225)	6.413954 (8.72327)	3.512015 (6.4310)	5.811728 (5.06051)	9.031083 (9.00190)	3.219355 (3.9414)	-0.29266 (2.48963)
*k*/*l*	19.2375 (7.69986)	26.72748 (16.4009)	7.48998 (8.7010)	35.0781 (11.5421)	49.70779 (24.7697)	14.62969 (13.228)	7.13971 (4.5266)
*scale*	16.42528 (14.9078)	44.47316 (46.1760)	28.04788 (31.268)	18.93467 (15.3854)	61.78672 (58.1105)	42.85205 (42.725)	14.80417 (11.457)

Note: The standard deviation is enclosed in parentheses.

The results show that the Beijing-Tianjin-Hebei Coordinated Industrial Development Policy improves fixed asset investment, capital deepening degree and enterprise scale, while decreases labor investment, R&D investment and number of enterprises. Since the implementation of the Beijing-Tianjin-Hebei coordinated development national strategy, the total amount of fixed asset investment in the region reached a historical peak of 5.48 trillion yuan in 2016, but in 2020, there was a rare negative growth, and the total amount of fixed asset investment in the whole society dropped to 5.32 trillion yuan. Since the reform and opening up, the total investment in The Beijing-Tianjin-Hebei region declined for the first time in 1989, with a decline of 8.8%. The second one was in 2017, with a year-on-year decline of 2.9%. The direct reason for the decline of the total investment in this region is that the total investment in Tianjin dropped significantly, which dropped 22.9% in 2017. The deep reason for Tianjin’s investment decline is the influence of environmental protection actions (closing tens of thousands of enterprises), economic structure adjustment and statistical system reform. In recent two years, Tianjin’s fixed asset investment showed a rapid recovery after the ‘water removal’. Due to the phased completion of the construction of Daxing airport and sub-center in Beijing, the investment in fixed assets dropped significantly. Hebei is benefited by the relocation of industrial enterprises from Beijing and the construction of Xiong’an New Area, and its fixed asset investment has maintained a steady trend. In order to relief the non-capital function of Beijing which is the core of this national strategy, the relocation of general manufacturing industries, especially those with high pollution, high energy consumption and emissions, directly leads to a decrease in the number of enterprises and the outflow of labor force. Among them, the labor force transfer from Beijing, which accounts for the largest proportion, does not occur in the Beijing-Tianjin-Hebei region, but to the Yangtze River Delta and the Pearl River Delta. Labor capital input is also shifting outward, so the policy effect of industrial coordination policy on the number of enterprises and labor input is not significant or even negative. R&D input has a significant promoting effect on industrial output growth and productivity improvement. However, the estimation results show that the industrial coordination policy has no promoting effect on R&D input. R&D input in the treatment group increased by 28.95% from 2007 to 2013, and plunged to 12.44% from 2014 to 2020, which is similar to that in the control group, and the R&D investment gap between regions narrowed sharply, with a differential result of -0.285. In terms of investment in innovation, the intensity of R&D investment in the Beijing-Tianjin-Hebei region increased by 0.51 percentage points from 3.48 percent in 2014 to 3.99 percent in 2020. The ratio of R&D investment intensity in Beijing, Tianjin and Hebei increased from 4.46:3.52:1 in 2014 (take Hebei as 1, the same below) to 3.68:1.97:1 in 2020, narrowing the gap between Hebei and Beijing and Tianjin. In terms of innovation output, the number of invention patents per 10,000 permanent residents in the region was 37 in 2020, an increase of 2.2 times over 2014. The turnover of regional technology market increased from 358.4 billion yuan in 2014 to 798.78 billion yuan in 2020, an increase of 1.2 times. In terms of innovation efficiency, 95.8 patents were granted per billion yuan of R&D funds in 2020, an increase of 61.8 percent compared with 2014.

It can be seen that the Beijing-Tianjin-Hebei Coordinated Industrial Development Policy is realized by increasing the capital, deepening degree of fixed asset investment and promoting the scale of enterprises, while the effect of labor input, R&D investment and the number of enterprises is not obvious.

### 3.3 Effect differentiation

Considering the Beijing, Tianjin, Hebei have their own different historical and industrial condition, the effect of coordinated development of three actual effect also have differences, so based on the analysis of the effect of policy in the previous section, it’s necessary to further explore the effect of the policy of Beijing-Tianjin-Hebei respectively. This part still uses the model to analyze the local policy effect of each region. The difference is that, in the analysis of effect differentiation, the data before 2014 is taken as the control group, and the data after 2014 (including) is taken as the processing group, and the difference method is used to estimate.

Based on the differential results in Table 7, it can be seen that:

**Table 7:** Differential results of GDP growth rate and total factor productivity in Beijing, Tianjin and Hebei.

Region Variable	Beijing	Tianjin	Hebei	Region Variable	Beijing	Tianjin	Hebei
*L*.*Δgdp*	0.1989*** (0.42)	-0.1339*** (-0.22)	0.6214*** (4.42)	*L*.*tfp*	-0.3238*** (-0.82)	-0.95152*** (-0.94)	0.2839*** (2.40)
*did*	0.0128*** (0.39)	-0.0317*** (-0.47)	0.0256* (1.49)	*did*	-0.0263*** (-0.42)	0.00869*** (0.08)	0.0158** (0.93)
*Δk*	0.2094*** (1.00)	0.0542*** (0.18)	-0.1137*** (-3.59)	*Δrd*	0.2842* (0.91)	0.06876 (0.54)	0.0474*** (2.39)
*Δl*	0.0785 (0.14)	0.2433 (0.59)	0.1420* (1.78)	*k*/*l*	0.1134*** (2.63)	0.00583* (1.80)	-0.0021*** (-2.38)
*Δrd*	0.2341 (1.05)	-0.0131 (-0.13)	0.0697*** (3.58)	*scale*	0.0010 (-1.07)	0.00194 (1.13)	0.0003 (0.53)
*num*	0.0002*** (0.11)	-0.0029*** (-0.52)	-0.0061* (-1.85)	*num*	-0.0025 (0.51)	0.00977 (0.63)	0.0068 (1.55)
*constant*	0.0076*** (0.07)	0.1765*** (0.83)	0.0306 (1.13)	*constant*	1.0366*** (2.99)	2.07704** (2.04)	0.8829*** (6.04)

First, the GDP growth rate of Beijing increased by 1.28%, while the total factor productivity decreased by 2.63. In terms of GDP growth rate, both capital growth rate and labor growth rate are significantly positive in Beijing. Under the stimulus of policy, which shows that the effect of capital input and labor input on driving industrial added value is quite obvious at the present stage. The growth rate of R&D expenditure is also significantly positive (0.23414), indicating that the current innovation input in Beijing has a significant driving effect on GDP growth rate; The number of enterprises coefficient is positive, which means that the number of enterprises in Beijing is basically stable in the process of industrial transfer, despite the existence of general manufacturing relief policy requirements. In the process of transfer, the leading enterprises with centralized enterprise scale and strong market power are retained, and the added value of production is strong, so it has a certain role in promoting the growth of industrial output. In the total factor productivity estimation, the coefficient of the growth rate of R&D expenditure and enterprise production scale are both positive, indicating that under the stimulation of enterprise innovation activities by the industrial collaborative development policy, innovation input has effectively promoted the reform of production mode and organization mode in Beijing, thus effectively improving industrial production efficiency. In addition, as a gathering place of large and medium-sized state-owned enterprises, Beijing’s remarkable advantages in scale economy make its policy effect more obvious; The coefficient of enterprise number is negative and not significant, which reflects to some extent that enterprise productivity is not directly affected by market competition brought about by industrial policies. The capital-labor ratio coefficient is significantly positive, indicating that capital deepening in Beijing has a promoting effect on enterprise productivity, and the benefits of capital input are considerable under the stimulus of policies.

Second, the GDP growth rate of Tianjin decreased by 3.17% overall, while the total factor productivity increased by 0.87. Specifically, in terms of GDP growth rate, both capital growth rate and labor growth rate in Tianjin are significantly positive, indicating that under the policy of industrial coordinated development, Tianjin has an obvious effect of driving industrial added value through capital input and labor input. As a national advanced manufacturing research and development base, Tianjin’s industrial structure is dominated by equipment manufacturing, automobile, petrochemical, aerospace and other manufacturing industries. Thanks to favorable policies, factor input has an immediate effect. The coefficient of growth rate of R&D expenditure is negative, indicating that the promotion effect of industrial coordinated development policy on Tianjin’s industrial innovation level is not obvious. As an old industrial city, Tianjin has a high proportion of traditional industries such as steel and petrochemical, which leads to great pressure of industrial transformation and low efficiency of innovation investment, especially insufficient transformation rate of achievements. In the short term, even if there are favorable policies, the contribution to positive growth will still take some time to appear. The coefficient of enterprise number is negative, which means that under the policy of relieving non-capital functions of Beijing, Tianjin has accepted some manufacturing enterprises from Beijing, but the number of enterprises in Tianjin has declined instead of rising. The reason is that under the requirements of the Beijing-Tianjin-Hebei coordinated environmental governance, Tianjin closed 30,000 small and dirty manufacturing enterprises and closed more than 200 industrial parks from 2016 to 2020. Scattered and dirty enterprises are those that do not conform to industrial policies and planning layout, illegal production and processing enterprises and storage enterprises, as well as zombie enterprises, empty shell enterprises. In addition, in the process of industrial transformation, the overall added value is low, which inhibits output growth. In total factor productivity estimation, the coefficient of the growth rate of research and development spending is positive, that illustrates under the stimulus of the coordinated development of industry policy, Tianjin industrial innovation strength are enhanced obviously. With the support of Beijing-Tianjin collaborative innovation and the construction of clusters such as China Xinchuang Valley, Cell Valley and Northern Sound Valley, will help promote the overall innovation level of the industry. Among them, Haihe Laboratory, as the core platform of industrial innovation, cooperates with multiple subjects such as leading enterprises, industrial alliances, incubation carriers, innovation platforms to build an innovation community and jointly carry out activities to train innovative talents for the transformation of scientific research achievements in the field of green creation and manufacturing of modern TCM materials, advanced computing and key software (ICT), synthetic biology and cell ecology. At present, Haihe Laboratory has gathered more than 3000 talents of various types led by 40 academicians of the Chinese Academies of Sciences and Chinese Academy of Engineering, from Beijing, Tianjin and Hebei province,100 national outstanding young scholars and Yangtze River scholars and attracted more than 30 leading enterprises to participate in it, effectively driving Tianjin’s industrial R&D investment and industrial transformation. The capital-labor ratio coefficient is significant and positive, indicating that the policy of coordinated industrial development accelerates the capital deepening degree of Tianjin. The key is that the coordinated development of Beijing, Tianjin and Hebei promotes the technological progress of Tianjin. On the one hand, technological progress can develop new products and open up new production service fields and new industries, thus creating new jobs. On the other hand, technological progress can improve labor productivity and reduce production costs, thus expanding production scale and increasing industrial demand for labor.

Third, the GDP growth rate of Hebei increased by 2.56% overall, and total factor productivity also increased by 1.58%. To be specific, in terms of GDP growth rate, under the incentive of industrial coordinated development policy, the growth rate of labor and R&D expenditure in Hebei are significantly positive, indicating that Hebei’s policy effect of driving industrial added value through labor input and R&D investment is relatively obvious. In the framework of the Beijing-Tianjin-Hebei Collaborative Development Strategy, the position of Hebei is the ‘important base of modern commercial logistics industry transformation, upgrading of the test area New type of urbanization, urban and rural areas as a whole demonstration area and Beijing-Tianjin-Hebei ecological environment support area’, thus, constitute ‘high and low’ collocation and dislocation development with Beijing’s positioning as a national political, economic and cultural center, Tianjin’s positioning as a research and development transformation and advanced manufacturing. The coefficient of capital growth rate and enterprise number is negative, which is mainly due to the low level of industrial structure in Hebei Province and the gap between Hebei and Beijing and Tianjin in industrial structure. Over the years, basic industries and traditional industries in Hebei province have a large proportion, the overall backward technology, equipment and technological level, lack of the development innovative technology industry, in the majority of industries are raw materials and initial product processing. As the two industries with the largest proportion in the industrial structure, thermoelectric production supply and metal smelting industry belongs to the resources industry. Therefore, even with the benefit of the industrial coordinated development policy, it is still difficult for Hebei to undertake the industrial division of labor from Beijing and Tianjin in the short term. In addition, the industrial transformation leads to the reduction of capital investment, and traditional enterprises begin to shrink. However, in the estimation of total factor productivity, the coefficient of growth rate of R&D expenditure is positive, the coefficient of number and size of enterprises is positive but not significant, and the coefficient of capital labor ratio is significantly negative, indicating that Hebei actively undertakes the industrial transfer from Beijing to Tianjin with the in-depth development of Beijing-Tianjin-Hebei coordinated development, and the pace of transformation and upgrading has accelerated. In 2019, the proportion of R&D expenditure in the GDP of Hebei province was 1.61%, ranking 16th in China, still lower than the national average level and significantly behind Beijing and Tianjin. There is room for further improvement in R&D investment. In 2019, the total income of high-tech enterprises in Hebei province accounted for 56.3% of the GDP, second only to Beijing, Shanghai, Guangdong, Tianjin and Zhejiang, which improved the situation that outdated production capacity was eliminated in large quantities and advanced production capacity was established slowly, but it was not obvious. In 2019, the ratio of market value to GDP of listed companies in Hebei province ranked 28th in China, lagging behind Beijing and Tianjin. It is difficult to trigger the reform of production mode and organization mode. A large amount of capital investment has resulted in low-efficiency capital enrichment, and the rapid decline of marginal income of capital factors has hindered the improvement of productivity.

In order to further explore the driving mechanism of policy effect differentiation within the Beijing-Tianjin-Hebei region, this section will explore the specific influence mechanism of various factor inputs, enterprise size and quantity changes on regional GDP growth rate and total factor productivity in Beijing, Tianjin and Hebei.

**Fig 2:**
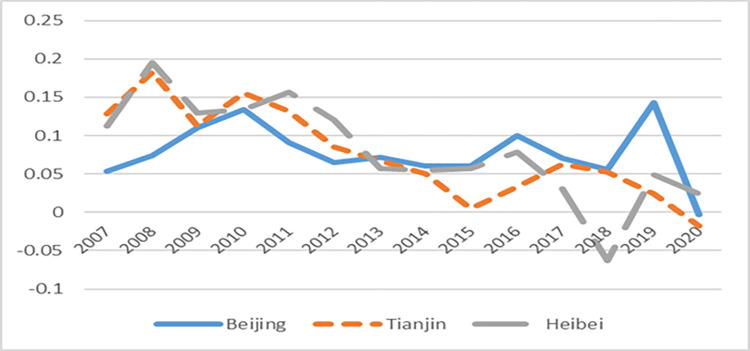
The trend of GDP growth rate in Beijing, Tianjin and Hebei.

**Fig 3:**
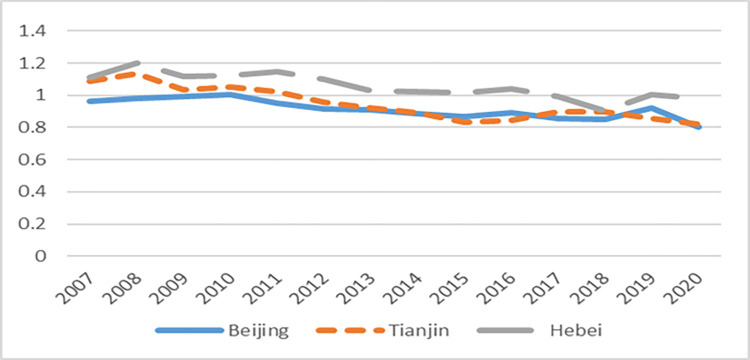
The trend of total factor productivity in Beijing, Tianjin and Hebei.

First of all, through the GDP growth rate and total factor productivity of Beijing-Tianjin-Hebei (Fig 2), (Fig 3) can find directly, on the one hand, the implementation of the coordinated development of industry policy has effectively promoted the convergence of the economic growth rate of Beijing, Tianjin and Hebei. Before 2014, the economic growth rate of Hebei and Tianjin was significantly higher than that of Beijing. In 2008, their economic growth rate lagged behind for about 1.5 years and began to decline continuously which was affected by the global economic crisis. After the implementation of the policy in 2014, the economic growth rates of the three regions basically kept pace, indicating that the implementation of the policy has played a role of community of destiny for the development of the regions. On the other hand, the total factor productivity of the region has always maintained a gentle trend of change, indicating that the implementation of the policy can improve the quality and efficiency of the Beijing-Tianjin-Hebei region, which also indicates that to some extent the improvement of total factor productivity is a long and slow process.

In order to further explore the internal mechanism of the local effects of industrial coordination policies in Beijing, Tianjin and Hebei, the dual difference method was used to investigate the effects of industrial coordination policies on each control variable in Beijing, Tianjin and Hebei.

As can be seen from Table 8, firstly, the implementation of the policy has a certain improvement effect on the capital input, labor input, R&D input and capital deepening degree in Beijing, while market competition and industrial scale have not improved. Second, the policy has an impact on Tianjin’s output and productivity mainly through increasing labor input, capital deepening and enterprise number growth, but has no obvious effect on capital input, R&D input and enterprise scale expansion. Third, the influence mechanism of the policy on Hebei’s output and productivity is mainly realized by increasing the investment in labor, R&D input, number of enterprises and expanding the scale of enterprises. Capital input has a driving effect on Hebei’s industrial total factor productivity, but does not promote the output growth in Hebei. At the same time, the capital deepening degree of Hebei has a negative effect on output and productivity, indicating that the Beijing-Tianjin-Hebei coordinated industrial development policy does not enhance the capital deepening degree of Hebei industry.

**Table 8:** Dual difference results of control variables in Beijing, Tianjin and Hebei.

variable	Beijing	Tianjin	Hebei
*Δk*	0.43317 (0.29388)	-0.41868 (0.52673)	0.01648 (0.03984)
*Δl*	0.68632 (1.10186)	0.70196 (0.57477)	0.31104 (0.07099)
*Δrd*	0.50828 (0.35188)	-0.21439 (0.12993)	0.07506 (0.02041)
*num*	-0.00047 (0.00181)	0.00737 (0.01212)	0.00849 (0.00243)
*k*/*l*	0.08746 (0.03494)	0.00737 (0.00378)	-0.00059 (0.00048)
*scale*	-0.00136 (0.00163)	-0.00012 (0.00150)	0.00180 (0.02402)

## 4 Discussion

It is difficult and crucial to judge whether industrial policy can promote economic growth and regional development. In this paper, Beijing, Tianjin and Hebei are taken as the treatment group and Shanxi, Shandong, Henan region are taken as the control group, uses the dual difference method and the panel data of prefecture-level cities from 2007 to 2020 to empirically study the policy effects of Beijing-Tianjin-Hebei industrial coordinated development and its implementation path from both quantitative and qualitative aspects. Taking 2014, when the policy was formally implemented, as the critical point, the data before 2014 was taken as the control group, and data after 2014 (including) was taken as the processing group to explore the local policy effects of the coordinated industrial development policies on The Beijing-Tianjin-Hebei region. The results show that the implementation of the Beijing-Tianjin-Hebei coordinated industrial development policy improves the total factor productivity of the region by 2.26% in quality, and reduces the GDP growth rate by 4.65 in quantity. Beijing’s GDP growth rate increased by 1.28%, and total factor productivity decreased by 2.63%. Tianjin’s GDP growth rate decreased by 3.17% and total factor productivity increased by 0.87%. Hebei’s GDP growth rate increased by 2.56%, and total factor productivity also increased by 1.58. The effect of the Beijing-Tianjin-Hebei coordinated industrial development policy is realized by increasing the capital deepening degree of fixed assets investment and promoting the scale of enterprises, while the effect of labor input R&D investment and the number of enterprises is not obvious.

At the same time, this paper finds some problems in the process of promoting collaborative policies, including the effect of industrial synergy policy on the growth rate of GDP added value is negative, the enhancement of capital deepening has a resistance to total factor productivity, the policy effect on the number of enterprises and labor input is not significant or even negative, industrial coordination policy does not promote R&D investment, etc. This shows that the implementation of the Beijing-Tianjin-Hebei industrial coordination policy has narrowed the inter-regional economic development gap within the region, but has no obvious effect on improving the regional economic development speed. A series of policy measures to promote coordinated development have strengthened input to the region, but the output has yet to be seen. At the same time, before and after the implementation of the policy, the total factor productivity, which reflects the "quality" change of economic growth in the region has little change, indicating that the source of economic growth in the Beijing-Tianjin-Hebei region still depends on factors driving.

The reason is that the traditional way of improving productivity by means of capital deepening has been reversed. A large amount of capital input has not changed its actual output with low efficiency, and the marginal income of capital input is decreasing year by year, which has produced resistance to the improvement of productivity. Although the intensity of innovation investment is reasonable, the transformation, application and industrialization of the achievements are not high, and the results of production mode organization and technological innovation are difficult to be reflected in a short time. The orderly removal of non-capital functions from Beijing is the core of the major national strategy for the coordinated development of the Beijing-Tianjin-Hebei region. General manufacturing in the Beijing-Tianjin-Hebei region, as well as enterprises with high pollution, high energy consumption and high emissions, have moved out of the region, directly leading to a reduction in the number of enterprises and the outflow of labor. The labor transfer in Beijing, which accounts for a large proportion, is not happening in the Beijing-Tianjin-Hebei region, but in the Yangtze River Delta and Pearl River Delta. Labor capital input is also shifting outward, leading to insignificant or even negative policy effect of industrial coordination policy on the number of enterprises and labor input. For each region, the effect of capital investment in R&D and labor investment in Beijing has already appeared, but its significance needs to be further enhanced. At present, Tianjin has low efficiency of innovation input and insufficient conversion rate of achievements. In the process of Tianjin and Hebei to undertake the industrial transfer from Beijing, the increase in the number of enterprises leads to fierce market competition, and the lack of agglomeration among many enterprises does not form economies of scale effect, and the overall industrial benefit is low. Especially, the capital utilization efficiency of Hebei is low, and a large number of capital investment has the phenomenon of low-efficiency capital density.

The policy implication reflected in the discussion is that to fully release the economic growth dividend of the Beijing-Tianjin-Hebei coordinated industrial development policy, we must pay equal attention to the stability and quantity of the Beijing-Tianjin-Hebei economic development. To be specific, first, increase investment in fixed assets in the Beijing-Tianjin-Hebei region, especially in "new infrastructure", increase investment in major projects, improve infrastructure and public service facilities, and drive regional economic development with high-quality and efficient investment. Second, speed up the integration of factor markets in the Beijing-Tianjin-Hebei region and fully release the advantages of human capital in Beijing and Tianjin. Third, continuing to strengthen R&D investment can stimulate the transformation of scientific and technological achievements through a series of preferential subsidies. In short, to achieve the strategic goal of coordinated industrial development in the Beijing-Tianjin-Hebei region, we need to focus on both quality and quantity. To keep making new progress, policy tools and implementation channels need to be constantly optimized.
